# Relationship of abstract thinking to mentalization in schizophrenia

**DOI:** 10.1192/j.eurpsy.2021.1407

**Published:** 2021-08-13

**Authors:** E. Sokolova, K. Andreyuk, A. Ryzhov

**Affiliations:** Faculty Of Psychology, Lomonosov MSU, Moscow, Russian Federation

**Keywords:** thought disorder, schizophrénia, mentalization

## Abstract

**Introduction:**

The formation of thinking in ontogenesis follows the line of progressive differentiation and integration of the representations of objects, events, and relationships. The same is true for the development of mentalization ability, conceived as a thought process in the area of social interactions.

**Objectives:**

The purpose of the study was to compare the particularities of thought processes when dealing with different types of material: physical objects (operational thinking) and social situations (mentalization).

**Methods:**

40 inpatients with schizotypal personality disorder, 40 inpatients with paranoid schizophrenia and 40 controls took part in the study. The Objects Sorting Test was used to assess operational thinking. The mentalization ability was assessed using two SCOR-S scales for Thematic Apperception Test: Complexity of representations of people and Understanding of social causality.

**Results:**

The results of correlation analysis support the existence of the reverse links between the impairments of operational thinking and both the complexity of representations of people (r=-.36, р<.001) and the understanding of social causality (r=-.38, р<.001). It is supported by the qualitative analysis, where inpatients with thought distortions, characterized by arbitrary generalizations, are inclined to make similar errors in the reasoning about the mental states, ignoring the conventional explanations and relying on their own emotional impression and etc.

**Conclusions:**

The limitations of the operational thinking as reflected in the inability to form adequate generalizations on the basis of socially predefined attributes of meaning are closely related to the ability of differentiation, integration and causal explanation of meaningful aspects or social situations.

